# Progesterone in the Brain: Hormone, Neurosteroid and Neuroprotectant

**DOI:** 10.3390/ijms21155271

**Published:** 2020-07-24

**Authors:** Rachida Guennoun

**Affiliations:** U 1195 Inserm and University Paris Saclay, University Paris Sud, 94276 Le kremlin Bicêtre, France; rachida.guennoun@inserm.fr

**Keywords:** progesterone, PR, allopregnanolone, neuroprotection, neurosteroid, stroke, traumatic brain injury, TBI

## Abstract

Progesterone has a broad spectrum of actions in the brain. Among these, the neuroprotective effects are well documented. Progesterone neural effects are mediated by multiple signaling pathways involving binding to specific receptors (intracellular progesterone receptors (PR); membrane-associated progesterone receptor membrane component 1 (PGRMC1); and membrane progesterone receptors (mPRs)) and local bioconversion to 3α,5α-tetrahydroprogesterone (3α,5α-THPROG), which modulates GABA_A_ receptors. This brief review aims to give an overview of the synthesis, metabolism, neuroprotective effects, and mechanism of action of progesterone in the rodent and human brain. First, we succinctly describe the biosynthetic pathways and the expression of enzymes and receptors of progesterone; as well as the changes observed after brain injuries and in neurological diseases. Then, we summarize current data on the differential fluctuations in brain levels of progesterone and its neuroactive metabolites according to sex, age, and neuropathological conditions. The third part is devoted to the neuroprotective effects of progesterone and 3α,5α-THPROG in different experimental models, with a focus on traumatic brain injury and stroke. Finally, we highlight the key role of the classical progesterone receptors (PR) in mediating the neuroprotective effects of progesterone after stroke.

## 1. Introduction

Steroid hormones are synthesized by adrenal glands, gonads, and placenta and influence the function of many target tissues including the nervous system. In addition, some steroids are synthesized de novo by neurons and glial cells and are called “neurosteroids” to refer to their site of synthesis: the nervous system [1]. Studies from several laboratories have shown the expression in the nervous system of the enzymes involved in steroidogenesis (reviewed in [2,3,4]). From an evolutionary point of view, data from different species demonstrate that neurosteroidogenesis is a conserved feature across fish, amphibians, birds, and mammals [2,5,6]. In addition to the novo synthesis of neurosteroids from cholesterol, some steroids can be locally converted in the nervous system to neuroactive metabolites, as in the case of progesterone which is metabolized into 3α,5α-tetrahydroprogesterone (3α,5α-THPROG; allopregnanolone). Neuroactive steroids designate all the steroids that can regulate neural functions including steroid hormones, neurosteroids, and synthetic steroids. Neuroactive steroids exert important functions in both the central and peripheral nervous systems and may represent a promising therapeutic strategy for the treatment of nervous system disorders [4,5,7].

Among neuroactive steroids, progesterone and its metabolite 3α,5α-THPROG have been extensively studied in brain, spinal cord, and sciatic nerve, and have been shown to regulate several functions including myelination, neuroprotection, and neuropathic pain [8,9,10,11,12,13]. This review aims to give an overview of the synthesis, metabolism, neuroprotective effects, and mechanism of action of progesterone in the rodent and human brain.

## 2. Progesterone Synthesis, Metabolism and Mechanism of Action in Brain

### 2.1. Progesterone Is an Endogenous Hormone and a Neurosteroid

Progesterone is a steroid hormone synthesized by ovaries and placenta in females and by adrenal glands in males and females. Thanks to its small size and lipid solubility, circulating progesterone easily crosses the blood brain barrier (BBB) by free transmembrane transport and diffuses throughout the nervous tissues [14,15,16,17]. However, most steroids bind and are transported by hormone-binding proteins in blood and this binding modulates their availability and their transport via the BBB. Concerning progesterone, this binding has a modest influence on its transport to the brain. Thus, 83% of the ^3^H-labeled progesterone was found in the ipsilateral rat brain hemisphere after 15 s post-injection in Ringer’s solution via the common carotid artery. This high transport of ^3^H progesterone via the BBB was not affected when the injection was in 67% rat serum and was slightly reduced by 18% when the bolus injection was in 67% human serum [14].

**Figure 1:**
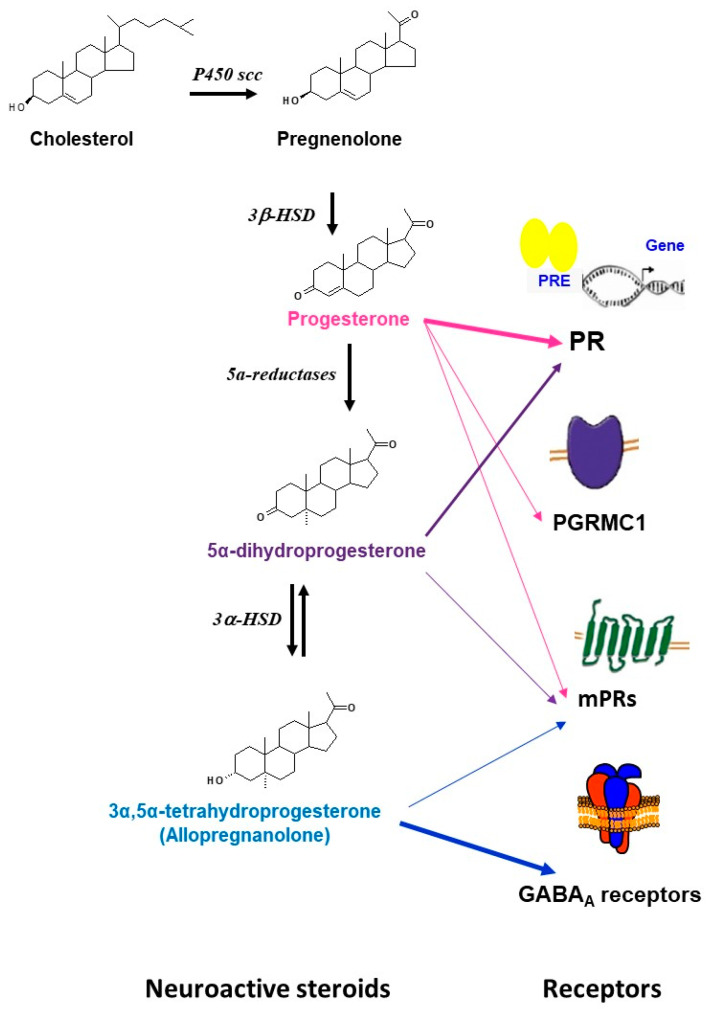
Schematic representation of the synthesis, metabolism, and main receptors that are involved in the actions of progesterone and its metabolites in brain. The precursor cholesterol is first converted into pregnenolone by the side chain cleavage cytochrome (P450scc) enzyme, pregnenolone is then converted to progesterone by the 3β-hydroxysteroid dehydrogenases (3β-HSD). Progesterone can further be bio-converted to 5α-dihydroprogesterone by 5α-reductases. 5α-dihydroprogesterone is converted to 3α,5α-tetrahydroprogesterone (allopregnanolone) by the 3α-hydroxysteroid oxidoreductase (3α- HSD) enzyme. Progesterone can bind to multiple receptors including the classical intracellular receptors (PR), the membrane receptors (mPRs: mPR α, mPβ, mPγ, mPRδ, mPRε), and the membrane-binding sites (PGRMC1). Some effects of progesterone may be mediated by its neuroactive metabolites. 5α-dihydroprogesterone binds to the classical receptors PR and has relatively high binding affinity for mPRα. 3α,5α-tetrahydroprogesterone (allopregnanolone) has no affinity for the intracellular PR, but is a potent allosteric modulator of GABA_A_ receptors. Some neuroprotective effects of allopregnanolone may be mediated by the membrane progesterone receptor mPRδ.

Progesterone is also a neurosteroid as it can be synthesized locally in the nervous system by glia and neurons [1,2,9,18,19,20,21]. Progesterone synthesis requires the conversion of cholesterol to pregnenolone by the cytochrome P450scc enzyme and then the conversion of pregnenolone to progesterone by the 3β-hydroxysteroid dehydrogenases (3β-HSD). Furthermore, progesterone can be sequentially converted to its neuroactive 5α-reduced metabolites: 5α-dihydroprogesterone (5α–DHPROG) by 5α-reductases; and 3α-5α-THPROG by 3α-hydroxysteroid dehydrogenases (3α-HSD) (Figure 1). Thus, the progesterone pool in the nervous system depends on (1) its peripheral synthesis, brain uptake, and accumulation; (2) its local synthesis; and (3) its metabolism.

### 2.2. Enzymes Involved in the Synthesis of Progesterone and Its Neuroactive Metabolites

The expression in the brain of the enzymes involved in progesterone synthesis and metabolism is well documented (For review see [2,3,4,9,20,22]).

P450scc expression was first detected by immunohistochemistry in the white matter of rat brain [23]. Subsequent studies detected P450scc expression in Purkinje cells in rat cerebellum [24,25] and pyramidal and granule cells in the hippocampus [26]. Cyp11A1 mRNA encoding P450scc was detected in different brain regions of rats with the highest level in the cerebral cortex [27]. In human brain, P450scc expression was first detected by immunohistochemistry in white matter [28]; and the expression of Cyp11A mRNA was detected in the neocortex, hippocampus, amygdala, caudate nucleus, cerebellum, corpus callosum, and thalamus [29,30,31]. Activity of the P450scc has been demonstrated in oligodendrocytes from rodents [32] and humans [33].

3β-HSD mRNA was detected in several regions of rat brain [22]. Using in situ hybridization, we showed a large distribution of 3β-HSD mRNA in particular in the olfactory bulb, striatum, cortex, thalamus, hypothalamus, septum, hippocampus, habenula, and cerebellum [34]. 3β-HSD mRNA expression was detected in different regions of human brain including the amygdala, caudate nucleus, corpus callosum, cerebellum, hippocampus, thalamus, and pons. The highest levels were measured in the corpus callosum [31,35]. Human oligodendroglial, astroglial, and neuronal cell lines in cultures express 3β-HSD [33]. 3β-HSD activity was demonstrated in rats using homogenates of amygdala and septum [36] and primary cultures of neurons, oligodendrocytes, and astrocytes [37,38,39,40,41,42].

5α-reductase 1 is the most abundant isoform of 5α-reductases expressed in the brain of mice, rats, and humans [43,44,45,46,47,48,49]. Recently, Giatti et al. showed that the expression of 5α-reductase mRNA in the rat cerebellum is higher in males compared to females [50]. 5α-reductase 1 mRNA was detected in the mouse brain in neurons of the olfactory bulb, cortex, hippocampus, amygdala, and cerebellum [51]. In humans, 5α-reductase 1 mRNA was detected in the cortex, hippocampus, cerebellum, hypothalamus, and pons [46,47,49]. In rat brain, 5α-reductase 1 was detected by immunohistochemistry in the hypothalamus, thalamus, hippocampus, cortex, and circumventricular organs [52,53]. At the cellular level, the 5α-reductase 1 immunoreactivity was described in glial and ependymal cells. 5α-reductase activity has been demonstrated in rat [54] and human brain [45,46,55]. Using rat neuronal cell cultures, Melcangi et al. demonstrated that neurons have a higher 5α-reductase activity than astrocytes and oligodendrocytes [56]. Interestingly, the activity of 5α-reductase in astrocytes is stimulated by co-culture with neurons or simply by addition of neuron-conditioned medium, suggesting stimulating diffusible factors from neurons [57]. One remarkable finding is the dramatic changes in steroidogenic activity observed during oligodendrocyte differentiation [42]. In particular, the activity of the 5α-reductase was five times higher in mature oligodendrocytes than in their pre-progenitors and their progenitors.

3α-HSD has been detected in the cortex, hippocampus, olfactory bulb, amygdala, and thalamus of both rat and mouse brain [51,58]. In rat cerebellum, the expression of 3α-HSD mRNA was higher in females compared to males and decreased after gonadectomy specifically in females [50]. 3α-HSD activity has been demonstrated in several regions of rat brain [58]. In neuronal cell cultures from rat brain, 3α-HSD activity was higher in astrocytes than in oligodendrocytes and neurons [56]. The investigation of progesterone metabolism at different stages of the oligodendrocyte lineage differentiation in vitro showed that 3α-HSD activity was ten times lower in the mature oligodendrocytes than in their pre-progenitors and progenitors [42]. mRNA of 3α-HSD type 2 and type 3 were detected in several regions of the human brain [22]. 3α-HSD activity has been demonstrated in cortex and white matter from biopsies from patients suffering from epilepsy [45].

It is important to note that the expression of the enzymes of neurosteroidogenesis has been reported to change under some pathological conditions. For example, there was a decrease in the 3β-HSD mRNA expression in the contusion site within the frontal cortex in both males and pseudopregnant female rats 24 h after traumatic brain injury (TBI) [59]. In a recent study, Leicaj et al. evaluated the expression of different enzymes of steroidogenesis in the brain of mice subjected to demyelination by cuprizone, a model of multiple sclerosis. They showed that the expression of P450scc and 5α-reductase was decreased in the hippocampus, cortex, and corpus callosum during the demyelination period and was restored to control levels during remyelination [60]. Using another model of multiple sclerosis, Noorbakhsh et al. have reported a significant decrease in 3α-HSD expression in the brain of autoimmune encephalomyelitis (EAE) mice [61]. Interestingly, studies in the Niemann–Pick type C (NP-C) mice, a model of fatal neurodegenerative human disease, showed a marked reduction in neurosteroidogenesis and suggest that this could contribute to the pathogenesis of the disease [62,63]. In fact, a reduced activity of 3α-HSD was observed in several brain regions of NP-C mice at birth. A reduced activity of 5α reductase was observed some weeks later before the onset of symptoms. In adult brains of NP-C mice, the expressions of P450scc, 3βHSD, 5α- reductase, and 3α-HSD were decreased in the cortex and cerebellum. In line with this, a dramatic decrease in the brain levels of 3α,5α-THPROG was reported in NP-C mice. Furthermore, symptoms were delayed by the administration of 3α,5α-THPROG [62]. In studies using postmortem human brain tissues, down-regulation of 5α-reductase type 1 and upregulation of 3α-HSD type 3 have been reported in substantia nigra of Parkinson’s disease patients [64]. In multiple sclerosis lesions, an up-regulation of 3β-HSD mRNA was observed in females but not in males [65]. In another study, a down-regulation of the transcripts of 5α-reductase type 1 and of 3α-HSD type 1 has been reported in the brains of multiple sclerosis patients [61].

### 2.3. Progesterone Receptors’ Expression in the Brain

Progesterone has multiple receptors and may activate several signaling pathways [9,20,21,66,67,68,69]. The first characterized are the intracellular receptors (PR). PR-A and PR-B are transcribed from two promoters of the same gene [70,71]. PRs act as nuclear transcription factors [9,70]. However, they have been also shown to interact with membrane-associated kinases and activate extranuclear signaling pathways [72]. In addition to PR, progesterone binds to the putative membrane-associated progesterone receptor component 1 (PGRMC1) that activates Jak/STAT, Src pathways, and protein kinase G [73,74,75,76]. Progesterone may also act after binding to the membrane progesterone receptors (mPR) that activate G-proteins and MAPK cascades. Five isoforms of mPR (mPRα, mPβ, mPγ, mPRδ, mPRε) encoded by distinct genes were cloned and characterized by Thomas and colleagues [77,78,79]. In addition, progesterone can be locally bio-converted to 3α,5α-THP (allopregnanolone) that modulates GABA_A_ receptors [9,80] (Figure 1). All these progesterone receptors are expressed and widely distributed in the brain and can account for the progesterone actions (for review see [9,20,66,68,69,73]).

It is important to note that after brain injury, changes in progesterone receptors’ expression have been observed. For example, we have shown that PGRMC1 was expressed in regions involved in cerebrospinal fluid production and in osmoregulation, and that its expression was increased in neurons and induced in astrocytes after traumatic brain injury (TBI) in both male and pseudopregnant female rats [81]. These observations suggest that PGRMC1 may be involved in the maintenance of water homeostasis and in decreasing edema formation after TBI. We have also shown that mPRα is expressed only by neurons in the absence of injury and that its expression is induced in microglia, astrocytes, and oligodendrocytes after TBI, suggesting its potential role in mediating the progesterone effects in inflammation, edema formation, and myelin repair after TBI [82]. After a transient cerebral ischemia, no significant changes in PR mRNA were noticed in the cortical penumbra obtained from cerebral cortex of male rats at 24h after ischemia [83]. However, another study showed an up-regulation of PR protein in the cytosol fraction prepared from the cortical penumbra at 24 and 48h post-ischemia [84]. After a permanent cerebral ischemia in rats, Stanojlovic et al. showed that levels of PR were decreased in both the cytosolic and nuclear fractions from the prefrontal cortex at 7 days post-injury and that progesterone treatment restored the PR levels to those observed in rats subjected to sham-operation [85]. Interestingly, a recent study in mice showed a decrease in PGRMC1 protein level in the cortical penumbra at day 7 following cerebral ischemia [86]. In studies using postmortem brain tissues, an up-regulation of PR expression was observed in multiple sclerosis lesions from female patients but not in male patients [65]. This difference may contribute to the observed gender differences in multiple sclerosis.

These observations provide examples indicating that progesterone signaling may be adjusted in response to injuries and thus it may differ according to physiological conditions and after brain injuries.

## 3. Brain Levels of Progesterone and Its Metabolites

### 3.1. Sex Differences and Decline during Aging

Effects of cycle, sex, and age on brain levels of progesterone and its metabolites are summarized in Table 1.

In mice, significant levels of progesterone, 5α–DHPROG, and 3α-5 α-THPROG were measured in brains of both young adult males and females [87,88,89,90]. In female mice, we have shown that brain levels of progesterone and 5α–DHPROG changed significantly according to estrus cycle [87]. Thus, brain levels of progesterone were in the same range or much higher in the brain of females compared to males depending on the phase of the cycle. In contrast, brain levels of 3α-5α-THPROG were not significantly different between males and females [87]. In rats, significant levels of progesterone, 5α–DHPROG, and 3α-5α-THPROG were measured in both young adult males and females [91,92]. Increased brain levels of progesterone, 5α–DHPROG, and 3α-5α-THPROG have been reported in pseudopregnant rats [91]. Evaluation of neurosteroid levels in different brain regions of male and female (at diestrus) rats showed subtle sex differences [92]. For instance, levels of progesterone in the cerebral cortex and the cerebellum were higher in males compared to females. In contrast, levels of 5α–DHPROG were higher in the hippocampus, cerebral cortex, and cerebellum in females compared to males. Levels of 3α-5α-THPROG were higher in the hippocampus and cerebral cortex in females compared to males [92]. Bixo and colleagues have measured the levels of 5α–DHPROG, and 3α-5α-THPROG in different regions of postmortem brain tissues from young and postmenopausal women. The highest levels of progesterone were measured in the hypothalamus, amygdala, and cerebellum; while the highest levels of 5α–DHPROG, and 3α,5α-THPROG were measured in the substantia nigra and hypothalamus [93].

Aging is associated with a decrease in steroid production. We have evaluated the profile of progesterone and its metabolites in the brain of young (3-month-old) and aged (20-month-old) male and female mice. Our data showed a strong decrease in brain levels of progesterone, 5α–DHPROG, and 3α-5α-THPROG in aged mice of both sexes. Interestingly, the sex differences observed in young mice were no longer observed in aged mice [87]. In rats, comparison of profiles in the limbic region of 7- and 24-month-old males showed decreased levels of progesterone and 3α-5α-THPROG but surprisingly increased levels of 5α–DHPROG [94]. Interestingly, analysis of postmortem human brain tissues showed that brain levels of progesterone, 5α–DHPROG, and 3α-5α-THPROG were higher in young women at the luteal phase of the cycle than in postmenopausal women suggesting a dependence of their brain levels on the synthesis in the periphery and a decline during aging [93].

### 3.2. Levels in Neurodegenerative Conditionsand in Response to Brain Injuries

Changes in brain levels of progesterone and its neuroactive metabolites were observed under neurodegenerative conditions and in response to brain injuries (Table 2).

Increased levels of 5α–DHPROG were measured in the limbic region of the brain of Alzheimer’s disease mice model 3xTg-AD mice compared to wild type littermates [94]. In contrast, decreased levels of 5α–DHPROG were measured in the striatum of male rats treated with 6-hydroxydopamine (an experimental model of Parkinson’s disease) [95], and in the cerebral spinal fluid of Parkinson’s disease patients [96]. Analysis of steroid levels in an experimental autoimmune encephalomyelitis (EAE) rat model, showed sex- and region-specific changes at the acute phase (14 days after immunization). For instance, in the cerebellum there was a decrease in progesterone in males and a decrease in 3α,5α-THPROG in females. In the cerebral cortex, there was a decrease in progesterone and 5α–DHPROG levels and an increase in 3α,5α-THPROG levels in males; while in females no changes were observed [97]. The same group has also measured the levels of steroids at the chronic phase of EAE (40 days post-immunization). They showed a decrease in progesterone, 5α–DHPROG, and 3α,5α-THPROG in the cerebral cortex of males and in the cerebellum of females [98]. Decreased levels of 3α,5α-THPROG were also reported in frontal lobe normal white matter near lesions obtained from male multiple sclerosis patients [61]. Levels of progesterone and its reduced metabolites were also altered after brain injuries. Thus, we have shown a transient increase in brain levels of progesterone and 5α–DHPROG, 6 h post-injury in male rats subjected to bilateral cortex contusion, a model of TBI. A transient increase of 5α–DHPROG level at 6 h post-injury, was also observed in pseudopregnant rat females that already had very high brain levels of progesterone and 5α–DHPROG at the time of injury. At 24 h post-injury, no significant differences were observed in both males and pseudopregnant females [91]. More recent studies investigated the potential dysregulation of steroids at 24 h, 72 h, and 2 weeks post-injury using the weight drop model of TBI in young male and female mice. In male mice, no differences were observed in brain levels at the three end points analyzed [89]. Surprisingly, decreased brain levels of progesterone, 5α–DHPROG, and 3α,5α-THPROG were measured in brain of female mice at the three times studied [88].

Unfortunately, there are no studies using post-mortem brain tissue from patients that died after TBI. However, a study using plasma from patients suffering from severe TBI showed a transient increase of progesterone levels specifically in men at day 0 post-injury, then a dramatic decrease from day 1 to day 6 post-injury in both men and women [99].

Changes in brain progesterone and 5α–DHPROG levels were also found in experimental stroke. For instance, using an experimental model of stroke by occlusion of the middle cerebral artery followed by reperfusion (MCAO/R) we showed an up-regulation of progesterone and 5α–DHPROG in both the contralateral and ipsilateral hemisphere of male mice at 6 h post-MCAO/R [100]. In a subsequent study, we performed a steroid profiling at different end points: 1, 2, 4, 6, and 24 h post-MCAO/R in both male and female mice [90]. Interestingly, in males we confirmed the increase at 6 h and showed that progesterone levels increased as early as 4 h, reaching a maximum at 6 h, then decreased to levels similar to controls at 24 h. Surprisingly, no significant variations were observed in these steroid levels in brains of female mice [90].

These observations provide examples indicating that brain levels of progesterone and its metabolites are dysregulated under pathological conditions and may be adjusted to respond to neurodegeneration and injuries. It is important to keep in mind that these regulations may be age-, sex-, and time-dependent. The decreases may reflect dysregulations associated with the pathologies and in this case normalization to control levels may have beneficial effects. On the other hand, increases may be part of endogenous neuroprotective and rescue processes and stimulation of the endogenous synthesis or exogenous administration of these steroids may optimize these natural responses.

## 4. Progesterone and Its Metabolites for Brain Neuroprotection

Progesterone has a broad spectrum of actions in the brain including reproduction and sexual behavior [101,102], CNS development and differentiation [103,104], myelination [105], and neuroprotection. In this review, we focus on the neuroprotective effects.

### 4.1. Neuroprotective Effects in Neurodegenerative Diseases Models

The neuroprotective effects of progesterone and its derivative 3α,5α-THPROG were demonstrated in different experimental models of neurodegenerative disease. For example, treatment of ovariectomized female 3xTg-AD mice (a model of Alzheimer’s disease) with progesterone alone or in combination with estradiol for 3 months specifically attenuated the hyperphosphorylation of Tau [106]. Extensive work by Brinton’s team focuses on the development of 3α,5α-THPROG as therapeutic for the treatment of Alzheimer’s disease. They showed in particular that 3α,5α-THPROG increased neurogenesis, improved cognitive function, and memory; decreased neuroinflammation and beta-amyloid accumulation; and restored the deficits of bioenergetics in the 3xTgAD mice [107,108,109,110]. Alzheimer’s disease patients at the early phase of the disease are now recruited for clinical trial-phase 1, to identify the effective dose of 3α,5α-THPROG (4-18 mg intramuscular weekly injections for 12 weeks) as a potential regenerative therapeutic for Alzheimer’s disease (Allopregnanolone regenerative therapeutic for early Alzheimer’s disease: intramuscular study (Allo-IM); Clinical Trials. Gov Identifier: NCT03748303).

In the case of Parkinson’s disease (PD), no definitive conclusion could be drawn from studies that investigated the therapeutic potential of progesterone using experimental models. Indeed, the results depend on the dose of progesterone, the treatment regimen, the sex, and the model used [111,112,113,114,115,116,117]. Interestingly, treatment by 3α,5α-THPROG improved cognitive and motor functions in Parkinson’s disease experimental models [118,119].

Importantly, progesterone showed efficient remyelinating and anti-inflammatory effects in different experimental models of multiple sclerosis and demyelination. For example, progesterone was shown to reduce the degeneration of oligodendrocytes, to increase the number of their progenitors, and to reduce astrogliosis, demyelination, and neurological deficits induced by cuprizone in male mice [120]. In female mice, progesterone and its synthetic derivative Nestorone, the high affinity selective ligand of PR, promoted myelin repair in cerebral cortex and corpus callosum with chronic demyelinating lesions induced by Cuprizone. In particular, progesterone increased the density of mature oligodendrocytes and their progenitors and decreased the density of activated astrocytes and microglia [121]. More recently, the beneficial effects of progesterone in this model were confirmed and extended with a focus on the anti-inflammatory effects [122]. This study showed that progesterone treatment decreased neurological deficits and demyelination; induced a switch in microglia phenotype from pro-inflammatory M1 to anti-inflammatory M2 type as shown by the expression of different specific markers; and decreased the NOD-like receptor pyrin domain containing 3 (NLRP3) inflammasome and the pro-inflammatory interferon-gamma inducing factor 18 (Il-18). In addition to the cuprizone model, the effectiveness of progesterone and 3α,5α-THPROG has been reported in the experimental autoimmune encephalomyelitis (EAE) model. In this model, progesterone showed promyelinating, neuroprotective, and anti-inflammatory effects, as recently reviewed [123]. In addition, the selective progesterone agonist Nestorone improved functional outcomes, increased neurogenesis, and decreased inflammation in the brains of female EAE mice [124]. In EAE mice, 3α,5α-THPROG limited axonal injury, demyelination, neuroinflammation, and functional deficits [61]. For more information regarding the effects of 3α,5α-THPROG on inflammation see [125,126]. For readers interested in the role of sex and sex hormones and their neuromodulatory effects and underlying mechanisms in the context of neurodegenerative diseases, we recommend two excellent recent reviews [127,128].

### 4.2. Neuroprotective Effects in TBI

There is an extensive literature reporting the neuroprotective effects of progesterone in experimental models of TBI. Briefly, progesterone treatment decreased edema, inflammation, BBB dysfunction, and promoted survival of newborn neurons and functional recovery (for review see [8,9,20,129,130,131,132,133]). Interestingly, 3α,5α-THPROG was also shown to be neuroprotective in experimental TBI. In particular, it decreased cell death, cognitive deficits, mitochondrial dysfunction, and inflammation [134,135,136,137]. Recruitment of patients will soon start for a phase II clinical trial (Allopregnanolone in chronic complex traumatic injury; Clinical Trials. Gov Identifier: NCT04003285). The trial aims to evaluate the potential of 3α,5α-THPROG (allopregnanolone) to improve depression, pain symptoms, and functional outcomes at several time points post-administration in patients with a mild traumatic injury.

Despite the multifactorial benefits of progesterone obtained in the experimental models of TBI and the promising results of two Phase II clinical trials [138,139], two Phase III clinical trials failed to show benefits of progesterone [140,141]. The possible reasons for this failure were recently discussed [142,143,144]. Among the concerns that have been raised we can cite: the high heterogeneity of the enrolled patients concerning sex, age, and severity of TBI; the high dose of progesterone used; the lack of stratification of patients; the subjective outcome measures; and the lack of follow up for a longer period. Several meta-analyses of the clinical trials are available [145,146,147,148]. Ma et al. included five randomized clinical trials. The results of the meta-analysis did not find evidence that progesterone could reduce mortality or disability. However, authors pointed to some inconsistencies and discussed potential bias that might reduce confidence in the conclusions. They recommend stratifying TBI patients and optimizing the dosage of progesterone for future trials [146]. The meta-analysis by Lu et al. (2016) included 8 clinical trials and did not reveal a significant effect of progesterone treatment in mortality or in neurological function in patients with acute TBI, and raised the same concerns [147]. The meta-analysis by Pan et al. included 8 clinical trials and showed that progesterone improved clinical outcomes in severe TBI at 3 months but not at 6 months [148].

In clinical trials, levels of progesterone in plasma of patients treated with progesterone were 100 times higher than the levels in patients treated with placebo; they reached a concentration of

1.1 µM [140,141,149], that is 10 to 1000 times higher than the Kd of progesterone receptors (PR: Kd = 1nM; mPRs: Kd = 10 nM; PGRMC1: Kd = 100 nM) [21]. The efficient cerebroprotective dose of progesterone (8 mg/kg) used in experimental rodent models of both TBI (Guennoun et al., unpublished results) and stroke [150] results in plasmatic levels of 150 nM, which is 10 times lower than the levels reached in patients treated with progesterone in the clinical trials (1.1µM). In the experimental models of stroke, progesterone brain levels reached 100 nM at 2 h after the last administration of progesterone [150]; these levels are compatible with the activation of PR (Kd = 1 nM); mPRs (Kd = 10 nM) and PGRMC1 (Kd = 100 nM). Depending on its brain concentration, progesterone may differentially activate its different receptors: higher doses of progesterone may saturate nuclear receptors (PR) while still activating membrane receptors mPR and PGRMC1. Higher doses might also induce the desensitization of receptors or decrease their expression, the saturation of pathways leading to neuroactive metabolites or the induction of inactivating metabolic pathways.

In summary, progesterone could be repurposed and repositioned in the treatment of TBI patients [151]. However, more pre-clinical studies with a better optimization of the formulation, dosing, and route of administration are needed [144]. It was also recommended to create a collaborative research network, to pool and more rigorously report preclinical studies, and to do more exploratory phase 2 trials [152]. Better definition of TBI and stratification according to sex, age, and severity, as well as development of biomarkers and better outcome measures are essential for future successful trials [133,142,143,147].

### 4.3. Neuroprotective Effects in Stroke Models

There is strong evidence that progesterone reduces the infarct volume and improves functional recovery in experimental models of stroke (see systematic reviews [153,154]). However, the meta-analysis by Wong et al. revealed that most studies used young male animals and none used gonadally intact young adult females. Different studies provided important information. For example, progesterone treatment decreased BBB disruption [150,155], hemorrhagic transformation [156], inflammatory response [84,157,158,159,160], mitochondrial dysfunction and oxidative damage [161,162,163,164,165], and apoptosis [166]. Moreover, progesterone has been reported to increase neurogenesis [167] and the survival of newborn neurons [168]. For further details see review [169]. Dose–response studies showed that the optimal neuroprotective dose is 8 mg/kg [170,171,172,173]. Of note, progesterone provides neuroprotection even when administered as late as 6 h after ischemia [171,174]. The observed transient increase of endogenous cerebral levels of progesterone after stroke [90,100] may provide an early endogenous neuroprotection that may contribute to this large therapeutic window.

Studies using 3α,5α-THPROG in ischemic stroke showed that it is efficient in reducing infarct size, BBB dysfunction, inflammation response, and behavioral deficits [155,163,175]. These findings led the authors to propose that the neuroprotective effects of progesterone may be mediated by its neuroactive metabolite 3α,5α-THPROG. However, our study using PR knock-out mice showed that although both treatments with progesterone or 3α,5α-THPROG are neuroprotective, PR play a key role in the mediation of the effect of progesterone [100].

## 5. Mechanism of Action of Progesterone after Stroke: A Key Role of PR

Our recent studies have revealed a key role of PR in neuroprotection after stroke. Thus, we have shown that at 6 h and 24 h post-MCAO, PR-dependent signaling of endogenous brain progesterone limited the extent of infarct size and the impairment of motor functions. Additional treatment with exogenous progesterone is required for optimal longer-term neuroprotection and is also PR-dependent [90,100,176]. Using total PR KO male mice, we first showed that PR expression is a limiting factor. Thus PR^−/−^ and even the heterozygous PR^+/−^ mice showed larger infarcts compared to wild type PR^+/+^ mice at 6 and 24 h post-MCAO [100]. We also showed the importance of PR after treatment with progesterone, as progesterone decreased infarct volume and motor deficits in wild type PR^+/+^ mice but not in PR^−/−^ mice [100]. Our findings, also demonstrated that the bio-conversion of progesterone to 3α,5α-THPROG is not the mechanism through which progesterone provides neuroprotection, otherwise progesterone treatment would have been neuroprotective in PR knockout (PR^−/−^) mice [100]. Another study confirms the importance of the level of expression of PR [177]. Indeed, progesterone decreased infarct size in wild type PR^+/+^ mice but did not in heterozygous mice PR^+/−^. To further clarify the role of PR specifically expressed in brain, we then generated a new transgenic mice line PR^NesCre^ mice in which PR expression was selectively invalidated in neural cells using the Cre-Lox strategy [90]. Invalidation of PR in neural cells resulted in increased brain tissue damage and neurological deficits in both male and female, young and aging mice at the early phase after stroke [90]. Interestingly, a greater effect was observed in young males compared to young females suggesting that females may have additional neuroprotective agents independent of PR. Using PR^NesCre^ mice and their control littermates PR^loxP/loxP^, we have also shown a key role of PR after progesterone treatment [176]. Thus, progesterone treatment improved motor coordination and reduced neurological deficits and infarct size in PR^loxP/loxP^ mice that express normal brain levels of PR but not in PR^NesCre^. At the cellular level, progesterone treatment increased the density of neurons, cells of the oligodendroglial lineage, and oligodendrocyte progenitors. Progesterone decreased the density of activated astrocytes and reactive microglia. These effects were observed in PR^loxP/loxP^ mice but not in PR^NesCre^ mice [176]. The key role of PR in neuroprotection was also shown by using Nestorone, the potent and selective PR agonist which does not interact with other receptors [178] and which is not bio-converted to GABA_A_-active metabolites [179]. We showed that, in male mice, Nestorone at very low dose decreased infarct volume and deficits in motor coordination [100]. Recently, a study by Tanaka et al. showed that the administration of Nestorone to male rats at 6 h post-MCAO reduced the infarct size at 48h post-MCAO and improved functional outcomes at long term (28 days post-MCAO) [180].

Although, there is strong evidence that PR is a major mediator of the neuroprotective effects of progesterone after cerebral ischemia, this does not exclude the involvement of additional mediators and cross talk with specific signaling pathways. The activation of these mechanisms may depend on the dose and timing of administration, on the time of analysis, and the outcomes measured. For example, Cai et al. showed that the activation of Src-ERK1/2 cascade via PR mediated the neuroprotective effects of progesterone observed at 48 h post-MCAO [181]. In addition, inhibiting the phosphoinositide 3-kinase/protein kinase B (PI3K/Akt) pathway, which regulates inflammation and cell survival, decreased the effects of progesterone observed on edema, infarct size, and VGEF levels at 24 h post-MCAO [166].

miRNAs (small noncoding RNAs that regulate gene expression) may also contribute to the neuroprotective effects of progesterone by acting post-transcriptionally to regulate the level of the mRNAs of the progesterone-related enzymes and receptors; progesterone may in turn regulate some miRNAs that are involved in cell death and inflammatory responses [68,182,183,184,185]. In the particular context of cerebral ischemia, a recent study in male mice showed a decrease in PGRMC1 protein level in the cortical penumbra at day 7 post-MCAO and that inhibition of let-7i miRNA alleviated the ischemia-induced decrease of PGMRC1 expression and increased the beneficial effects of progesterone [86]. Recently, Herzog et al. investigated the role of estradiol and progesterone on the regulation of selected miRNA and the genes they regulate. They showed a time-dependent increase in the levels of specific miRNAs in the peri-infarct; and that progesterone like estradiol decreased the levels of miR-223 and mi-214 and increased the level of mi-375. Furthermore, progesterone regulated the related target genes of miR-223 and miR-375 in the peri-infarct at 24 h post-MCAO. Thus, this indirect control of the genes involved in apoptosis and inflammation may contribute to the neuroprotective effects of progesterone after stroke [186].

## 6. Summary and Concluding Remarks

**Figure 2:**
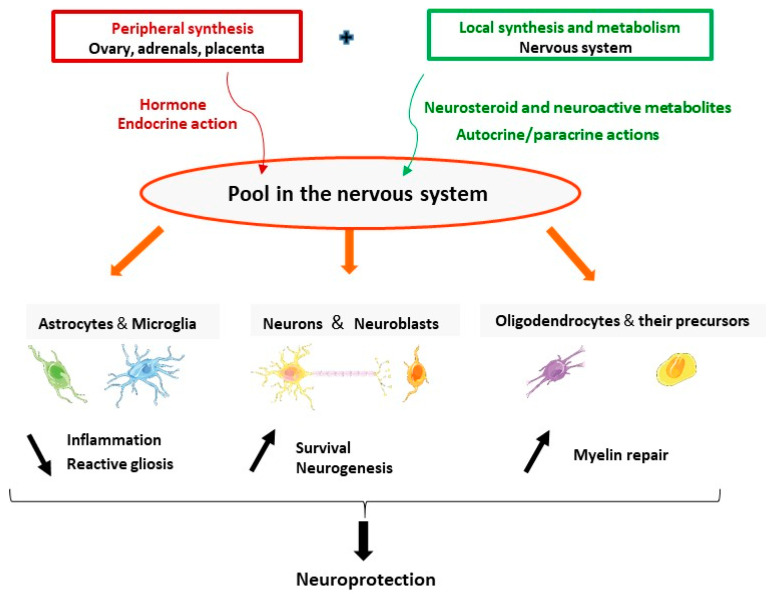
The brain is a site of synthesis, metabolism, and action of progesterone. Progesterone (either synthesized in the endocrine glands or locally in the nervous system) and its neuroactive metabolites have pleiotropic neuroprotective effects. They act in astrocytes and microglia to reduce inflammation and reactive gliosis; promote survival of neurons and neurogenesis; and increase myelination by acting in oligodendrocytes and their precursors.

The brain is a site of synthesis, metabolism, and action of progesterone. Progesterone and its neuroactive metabolites have pleiotropic protective effects in neurons and glial cells including reduction of inflammation and reactive gliosis; and promoting neuroprotection and neurogenesis and myelin repair (Figure 2). Synthesis and actions of progesterone may be adjusted on demand, to respond to brain injuries and may be affected by neurodegenerative diseases. The stimulation of neurosteroidogenesis may be part of the endogenous neuroprotective processes. One therapeutic strategy is to reinforce these intrinsic natural mechanisms by pharmacological treatments. The data reviewed here give a strong hint that treatment by progesterone has a great therapeutic potential as a protective agent to reduce brain damage. The failure of the clinical trials using progesterone for TBI patients pointed to many aspects that should be considered and revised. Progress in understanding the mechanisms of action of progesterone in specific pathological conditions will help to better design future clinical trials. In the particular case of stroke, the identification of PR as a major mediator of neuroprotection indicates that PR agonists may also represent a potential efficient treatment.

## Figures and Tables

**Table 1:** Brain levels of progesterone (PROG), 5α-dihydroprogesterone (5α-DHPROG), and 3α,5α-tetrahydroprogesterone (3α,5α-THPROG): effect of cycle, sex, and age.

Steroid	Cycle Effect	Sex Effect	Age Effect	Species/Structures	References
PROG	D > P > E	F (D) > M	Decrease	Mice/Cerebral hemisphere	[87]
5α-DHPROG	D > P > E	NS	Decrease		
3α,5αTHPROG	NS	NS	NS		
PROG		NS		Rats/Hippocampus	[92]
		M > F (D)		Rats/Cortex, Cerebellum	
5α-DHPROG		F (D) > M		Rats/Hippocampus, Cortex, Cerebellum	
3α,5α-THPROG		F (D) > M		Rats/Hippocampus, Cortex	
PROG			Decrease	Rats/Limbic regions	[94]
5α-DHPROG			Increase		
3α,5α-THPROG			Decrease		
PROG			Decrease	Human/Cortex, Amygdala, Hippocampus, Striatum, Thalamus	[93]
5α-DHPROG			Decrease		
3α,5α-THPROG			Decrease		

Sex effect: 3-month-old male mice vs. 3-month-old female mice in diestrus stage. Age effect: 20-month-old mice vs. 3-month-old mice of both sexes; 24-month-old male rats vs. 7-month-old male rats; postmenopausal (59–75 years) women vs. fertile women (18–38 years) at the luteal phase. Stages of the estrus cycle: proestrus (P); estrus (E); diestrus (D). F: females; M: males; NS: not significant.

**Table 2:** Levels of progesterone (PROG), 5α-dihydroprogesterone (5α-DHPROG), and 3α,5α-tetrahydroprogesterone (3α,5α-THPROG) under neurodegenerative conditions and in response to brain injuries.

Pathology/Experimental Model	Sex/Species	Steroids/Change	Structures	References
**AD/3xTg-AD mice**	Male mice	5α-DHPROG ↑	Limbic region	[94]
**P/ 6-OHDA**	Male rats	5α-DHPROG ↓	Cortex, Striatum	[95]
**PD**	Men	5α-DHPROG ↓	CSF	[96]
**EAE acute phase**	Male rats	PROG ↓	Cerebellum	[97]
		PROG ↓ 5α-DHPROG ↓ 3α,5α-THPROG ↑	Cortex	
	Female rats	3α,5α-THPROG ↓	Cerebellum	
**EAE chronic phase**	Male and female rats	PROG ↓ 5α-DHPROG ↓ 3α,5α-THPROG↓	Cortex of males Cerebellum of females	[98]
**MS**	Men	3α,5α-THPROG ↓	Frontal lobe	[61]
**TBI/Bilateral cortex contusion**	Male rats	PROG ↑ 5α-DHPROG ↑ (6 h post-TBI)	Brain	[91]
	Pseudopregnant Female rats	5α-DHPROG ↑ (6 h post-TBI)		
	Male mice	PROG = 5α-DHPROG = 3α,5α-THPROG =(24 h, 72 h, 2 weeks post-TBI)	Brain	[89]
**TBI/weight drop model**	Female mice	PROG ↓ 5α-DHPROG ↓ 3α,5α-THPROG ↓ (24 h, 72 h, 2 weeks post-TBI)	Brain	[88]
**Severe TBI**	Men	PROG ↑ (Day 0 post-TBI)	Plasma	[99]
	Men and Women	PROG ↓ (Days 1 to 6 post-TBI)		
**Stroke MCAO/R**	Male mice	PROG ↑ (4 h, 6 h post-MCAO)	Cerebral hemisphere	[90]
		5α-DHPROG ↑ 3α,5α-THPROG = (1,2,4,6, 24 h post-MCAO)		
	Female mice	PROG = 5α-DHPROG = 3α,5α-THPROG = (1,2,4,6, 24 h post-MCAO)		

6-OHDA: 6-hydroxydopamine; AD: Alzheimer’s disease; CSF: cerebrospinal fluid; EAE: Experimental autoimmune encephalomyelitis; MCAO/R: Middle cerebral artery occlusion/reperfusion; MS: multiple sclerosis; PD: Parkinson’s disease; TBI: traumatic brain injury. ↑ Increase; ↓ Decrease; = No change.

## Abbreviations

3α-HSD	3α-hydroxysteroid dehydrogenase
3β-HSD	3β-hydroxysteroid dehydrogenase
5α-DHPROG	5α-dihydroprogesterone
3α,5α-THPROG	3α,5α-tetrahydroprogesterone (allopregnanolone)
6-OHDA	6-hydroxydopamine
AD	Alzheimer’s disease
BBB	Blood brain barrier
CNS	Central nervous system
CSF	Cerebrospinal fluid
EAE	Experimental autoimmune encephalomyelitis
GABA	γ-aminobutyric acid
Il-18	Interferon-gamma inducing factor 18
MCAO/R	Middle cerebral artery occlusion/reperfusion
mPR	Membrane progesterone receptors
MS	Multiple sclerosis
NLRP3	NOD-like receptor pyrin domain containing 3
NP-C	Niemann–Pick type C
P450scc	Cholesterol side-chain cleavage
PD	Parkinson’s disease
PGRMC1	Membrane-associated progesterone receptor membrane component 1
PR	Progesterone receptors
PROG	Progesterone
TBI	Traumatic brain injury
